# Gene Recruitment of the Activated *INO1* Locus to the Nuclear Membrane

**DOI:** 10.1371/journal.pbio.0020342

**Published:** 2004-09-28

**Authors:** Jason H Brickner, Peter Walter

**Affiliations:** **1**Howard Hughes Medical Institute and the Department of Biochemistry and Biophysics, University of CaliforniaSan Francisco, CaliforniaUnited States of America

## Abstract

The spatial arrangement of chromatin within the nucleus can affect reactions that occur on the DNA and is likely to be regulated. Here we show that activation of *INO1* occurs at the nuclear membrane and requires the integral membrane protein Scs2. Scs2 antagonizes the action of the transcriptional repressor Opi1 under conditions that induce the unfolded protein response (UPR) and, in turn, activate *INO1*. Whereas repressed *INO1* localizes throughout the nucleoplasm, the gene is recruited to the nuclear periphery upon transcriptional activation. Recruitment requires the transcriptional activator Hac1, which is produced upon induction of the UPR, and is constitutive in a strain lacking Opi1. Artificial recruitment of *INO1* to the nuclear membrane permits activation in the absence of Scs2, indicating that the intranuclear localization of a gene can profoundly influence its mechanism of activation. Gene recruitment to the nuclear periphery, therefore, is a dynamic process and appears to play an important regulatory role.

## Abstract 2

A study of the yeast gene *INO1* indicates that the recruitment of the gene to the nuclear membrane appears to play an important part in its regulation

## Introduction

For over a hundred years, it has been recognized that chromatin is distributed non-randomly within the interphase nucleus (Rabl 1885; Boveri 1909). More recently, three-dimensional fluorescence microscopy studies have established that chromosomes are organized into distinct, evolutionarily conserved subnuclear territories (reviewed by Cockell and Gasser [1999]; Isogai and Tjian [2003]). However, DNA is mobile and can move between these domains (reviewed in Gasser [2002]). Recent studies suggest that the subnuclear localization of genes can have dramatic effects on their chromatin state, rate of recombination, and transcription (Cockell and Gasser 1999; Isogai and Tjian 2003; Bressan et al. 2004). Heterochromatin, for example, is generally found concentrated in close proximity to the nuclear envelope. Several genes conditionally colocalize with heterochromatin under conditions in which they are repressed. The transcriptional regulator Ikaros, for example, interacts both with regulatory sequences upstream of target genes and with repeats enriched at centromeric heterochromatin. When repressed, these genes become colocalized with heterochromatin, suggesting that Ikaros promotes repression by directly recruiting target genes into close proximity with heterochromatin (Brown et al. 1997, 1999; Cobb et al. 2000). Consistent with this view, euchromatic sequences that become colocalized with heterochromatin are transcriptionally silenced (Csink and Henikoff 1996; Dernburg et al. 1996).

In *Saccharomyces cerevisiae,* genes localized in proximity to telomeres are similarly transcriptionally silenced (Gottschling et al. 1990). Silencing is due to Rap1-dependent recruitment of Sir proteins to telomeres (Gotta et al. 1996), which promotes local histone deacetylation and changes in chromatin structure (reviewed in Rusche et al. [2003]). Physical tethering of telomeres at the nuclear periphery through interactions with the nuclear pore is required for silencing (Gotta et al. 1996; Laroche et al. 1998; Galy et al. 2000; Feuerbach et al. 2002). When a reporter gene flanked by silencer motifs was relocated more than 200 kb away from a telomere, silencing was lost (Maillet et al. 1996). Silencing was restored to this gene by overexpression of *SIR* genes. Therefore it is thought that tethering serves to promote efficient recruitment of Sir proteins, which are enriched at the nuclear periphery and limiting elsewhere (Maillet et al. 1996). Another example of gene silencing at the nuclear periphery comes from experiments in which defects in the silencer of the *HMR* locus could be suppressed by artificially tethering this locus to the nuclear membrane (Andrulis et al. 1998). Thus, localization of chromatin to the nuclear periphery has been proposed to play a major role in transcriptional repression.

By contrast, we report here that dynamic recruitment of genes to the nuclear membrane can have profound effects on their activation. The gene under study here is *INO1,* a target gene of the unfolded protein response (UPR), which encodes inositol 1-phosphate synthase. The UPR is an intracellular signaling pathway that is activated by the accumulation of unfolded proteins in the endoplasmic reticulum (ER), which can be stimulated by treatment with drugs that block protein folding or modification or, in yeast, by starvation for inositol (Cox et al. 1997). These conditions activate Ire1, a transmembrane ER kinase/endoribonuclease (Cox et al. 1993; Mori et al. 1993), which, through its endonuclease activity, initiates nonconventional splicing of the mRNA encoding the transcription activator Hac1 (Cox and Walter 1996; Shamu and Walter 1996; Kawahara et al. 1997; Sidrauski and Walter 1997). Only spliced *HAC1* mRNA is translated to produce the transcription factor; the Ire1-mediated splicing reaction, therefore, constitutes the key switch step in the UPR (Sidrauski et al. 1996; Ruegsegger et al. 2001).

Hac1 is a basic-leucine zipper transcription factor that binds directly to unfolded protein response elements (UPREs) in the promoters of most target genes to promote transcriptional activation (Cox and Walter 1996; Travers et al. 2000; Patil et al. 2004). However, a subset of UPR target genes uses a different mode of activation. Transcriptional activation of these genes, including *INO1,* depends on Hac1 and Ire1. These target genes contain an upstream activating sequence that is regulated by the availability of inositol, the UASINO element, in their promoters that is repressed by Opi1 under non-UPR conditions (Greenberg et al. 1982; Cox et al. 1997). Opi1 repression is relieved in a Hac1-dependent manner upon induction of the UPR (Cox et al. 1997). Positively acting transcription factors Ino2 and Ino4 then promote transcription from UASINO-containing promoters (Loewy and Henry 1984; Ambroziak and Henry 1994; Schwank et al. 1995). Our previous work established that the production of Hac1 by UPR induction functions upstream of Opi1, suggesting that the role of the UPR is to counteract Opi1-mediated repression (Cox et al. 1997).

To understand the regulation of UASINO-controlled genes by the UPR, we have examined the molecular events leading to the activation of *INO1*. We find that Scs2, an integral protein of the nuclear and ER membrane that was recently shown to play a role in telomeric silencing (Craven and Petes 2001; Cuperus and Shore 2002), is required to activate *INO1*. We observe dynamic *INO1* recruitment to the nuclear membrane under activating conditions. Importantly, we find that recruitment requires Hac1 and is opposed by Opi1. Furthermore, we show that artificial recruitment of *INO1* to the nuclear membrane can bypass the requirement for Scs2. Gene recruitment to the nuclear membrane therefore plays an instrumental role in *INO1* activation.

## Results

### Abundance and Localization of the Transcriptional Regulators Ino2, Ino4, and Opi1 Are Unaffected by UPR Induction

**Figure 1:**
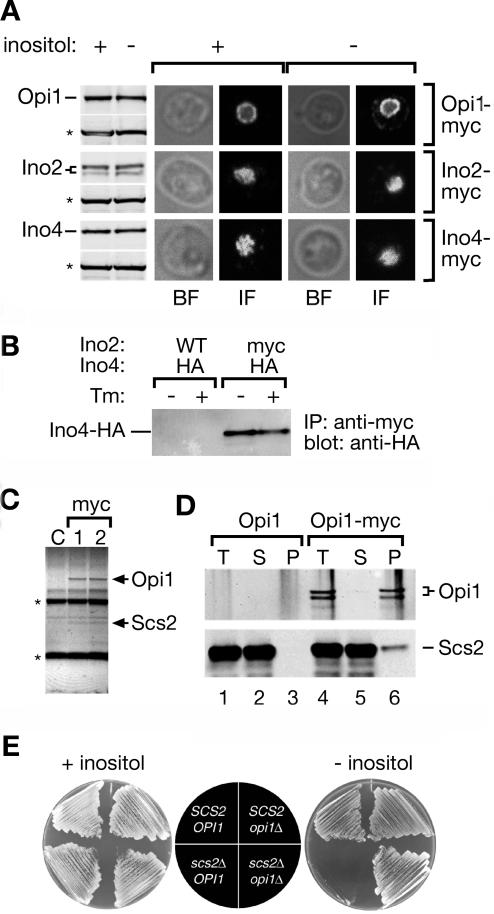
Scs2 Regulates the Function of Opi1 on the Nuclear Membrane (A) Steady state protein levels and localization of Opi1, Ino2, and Ino4 under repressing and activating conditions. Strains expressing *myc*-tagged Opi1, Ino2, or Ino4 (Longtine et al. 1998) were grown in the presence (*INO1* repressing condition) or absence (*INO1* activating condition) of *myo*-inositol for 4.5 h. Tagged proteins were analyzed by Western blotting (size-fractionated blots on the left, designated Opi1, Ino2, and Ino4) and indirect immunofluorescence (photomicrographs on the right). For Western blot analysis, 25 μg of crude lysates were immunoblotted using monoclonal antibodies against either the *myc* epitope (top bands in each set) or, as a loading control, Pgk1 (bottom bands in each set; indicated with an asterisk). Immunofluorescence experiments were carried out using anti-*myc* antibodies and anti-mouse Alexafluor 488. Bright-field (BF) and indirect fluorescent (IF) images for a single z slice through the center of the cell were collected by confocal microscopy. (B) Ino2 and Ino4 heterodimerize under both repressing and activating conditions. Cells expressing either HA-tagged Ino4 (negative control) or HA-tagged Ino4 and *myc*-tagged Ino2 were grown in the presence or absence of 1 μg/ml tunicamycin (Tm; an inhibitor of protein glycosylation that induces protein misfolding in the ER) for 4.5 h and lysed. Proteins were immunoprecipitated using the anti-*myc* monoclonal antibody. Immunoprecipitates were size-fractionated by SDS-PAGE and immunoblotted using the anti-HA monoclonal antibody. (Continued on next page) (C) Coimmunoprecipitation of Scs2 with Opi1. Detergent-solubilized microsomal membranes from either an untagged control strain (lane C) or duplicate preparations from the Opi1-*myc* tagged strain (*myc* lanes, 1 and 2) were subjected to immunoprecipitation using monoclonal anti-*myc* agarose. Immunoprecipitated proteins were size-fractionated by SDS-PAGE and stained with colloidal blue. Opi1-*myc* and the band that was excised and identified by mass spectrometry as Scs2 are indicated. IgG heavy and light chain bands are indicated with an asterisk. (D) Coimmunoprecipitation with tagged proteins. Immunoprecipitation analysis was carried out on strains expressing either Scs2-HA alone (lanes 1–3) or Scs2-HA together with Opi1p-*myc* (lanes 4–6). Equal fractions of the total (T), supernatant (S), and bound (B) fractions were size-fractionated by SDS-PAGE and immunoblotted using anti-*myc* or anti-HA monoclonal antibodies. (E) Epistasis analysis. Haploid progeny from an *OPI1/opi1*Δ*SCS2/scs2*Δ double heterozygous diploid strain having the indicated genotypes were streaked onto minimal medium with (+ inositol) or without (– inositol) 100 μg/ml *myo*-inositol and incubated for 2 d at 37 °C.

To characterize the molecular basis of transcriptional activation of *INO1,* we first asked whether the steady-state levels of the known transcriptional regulators—the activators Ino2 and Ino4 and the repressor Opi1—were affected by induction of the UPR. To this end, we monitored the levels of *myc*-tagged proteins by Western blotting after UPR induction by inositol starvation (Figure 1A). Induction of the UPR did not result in a significant change of the abundance of any of the proteins. Thus, in contrast to what has been suggested in previous studies (Ashburner and Lopes 1995a, 1995b; Cox et al. 1997; Schwank et al. 1997; Wagner et al. 1999), *INO1* transcription is not regulated through adjustment of the abundance of these regulators.

Next, we tested whether the subcellular localization of these regulators is modulated. We examined the localization of *myc*-tagged Opi1, Ino2, and Ino4 by indirect immunofluorescence (Figure 1A). Again, we observed no significant change upon UPR induction: Ino2 and Ino4 localized to the nucleus under both repressing and activating conditions. Localization of Opi1 also showed no change. Like Ino2 and Ino4, Opi1 localized to the nucleus under both conditions. However, in agreement with recent data by Loewen et al. (2003), we found that Opi1 was concentrated at the nuclear membrane and diffusely distributed throughout the nucleoplasm (Figure 1A). Furthermore, coimmunoprecipitation experiments showed that Ino2 and Ino4 heterodimerize under both conditions, suggesting that this interaction is not regulated (Figure 1B). Taken together, these observations therefore pose an interesting puzzle: How is regulation achieved when the localization and abundance of all three regulators is unchanged between activating and repressing conditions?

### Opi1 Is Regulated by an Integral ER/Nuclear Membrane Protein

To begin to explore a possible functional significance of Opi1's unusual localization pattern at the nuclear membrane, we sought to identify binding partners that might tether Opi1 to the membrane. To this end, we immunoprecipitated *myc*-tagged Opi1 under nondenaturing conditions from mildly detergent-solubilized microsomal membranes. Bands that were enriched in the immunoprecipitated fraction from the *myc*-tagged strain were identified by matrix-assisted laser desorption ionization mass spectrometry (Figure 1C). This procedure identified Scs2, a bona fide integral membrane protein known to reside in nuclear membranes and ER (Nikawa et al. 1995; Kagiwada et al. 1998; Kagiwada and Zen 2003). To confirm that Scs2 and Opi1 interact, we performed coimmunoprecipitation analysis from extracts of strains expressing *myc*-tagged Opi1 and hemagglutinin (HA)-tagged Scs2. We observed specific recovery of Scs2-HA in Opi1-*myc* immunoprecipitates (Figure 1D). Recent results from a genome-wide immunoprecipitation study (Gavin et al. 2002) and in vitro peptide binding studies (Loewen et al. 2003) corroborate the interaction between Opi1 and Scs2.

In contrast to Opi1, the transcriptional repressor Scs2 has been implicated in the activation of *INO1* transcription: Overexpression of *SCS2* suppresses the Ino^–^ growth phenotype in cells that cannot activate the UPR (Nikawa et al. 1995), and loss of Scs2 impairs activation of *INO1* (Kagiwada et al. 1998; Kagiwada and Zen 2003). Therefore, either Scs2 is the downstream target of Opi1-mediated repression, or Scs2 functions upstream to relieve Opi1-mediated repression. To distinguish between these possibilities, we analyzed the growth of the double mutant in the absence of inositol. As shown in Figure 1E, *opi1*Δ cells grew in absence of inositol because *INO1* is constitutively expressed. In contrast, *scs2*Δ cells did not grow under these conditions. Double mutant *opi1*Δ *scs2*Δ cells grew in the absence of inositol, indicating that Scs2 functions to regulate Opi1 and is dispensable in the absence of Opi1. Given that Scs2 is an integral membrane protein, these data suggest that regulation of Opi1 occurs at the nuclear membrane.

### Ino2 and Ino4 Bind to the *INO1* Promoter Constitutively

**Figure 2:**
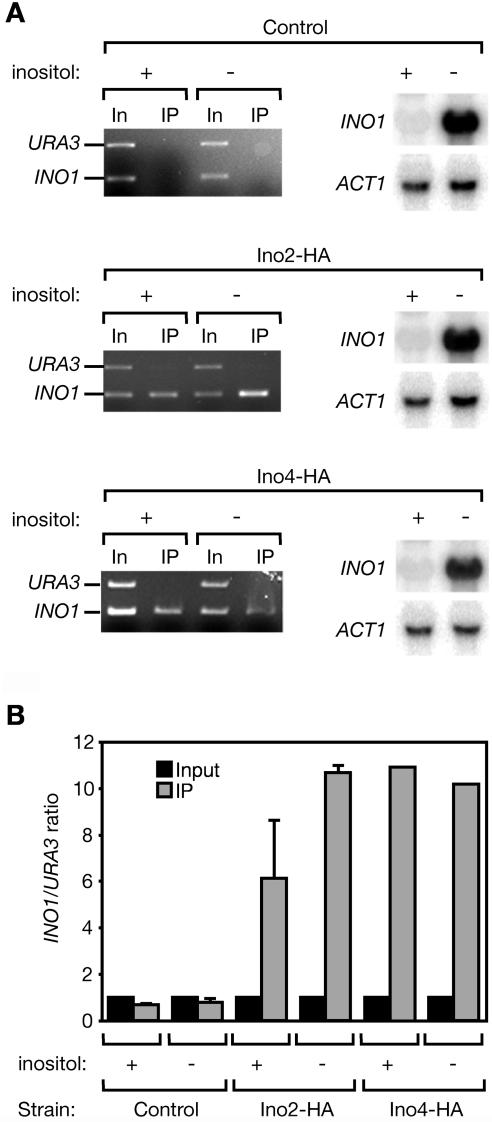
Ino2/Ino4 Bind to the *INO1* Promoter Constitutively (A) Untagged control cells (upper images), or cells in which the endogenous copies of *INO2* and *INO4* were replaced with HA-tagged Ino2 (center images) or HA-tagged Ino4 (lower images) were harvested in mid-logarithmic phase and washed into medium with or without *myo*-inositol. After 4.5 h, about 1.5 × 10^8^ cells were harvested and processed for Northern blot analysis (light images with dark bands, right). Northern blots were probed against both *INO1* and *ACT1* (loading control) mRNA. The remaining cells were fixed with formaldehyde and lysed. Chromatin was sheared by sonication and then subjected to immunoprecipitation with anti-HA agarose. Input DNA (In) and immunoprecipitated DNA (IP) were analyzed by PCR using primers to amplify the *INO1* promoter and the *URA3* gene. Amplified DNA was size-fractionated by electrophoresis on ethidium bromide-stained agarose gels (dark images with light bands, left). (B) Quantitative PCR analysis. Input and IP fractions were analyzed by real-time quantitative PCR. The ratio of *INO1* promoter to *URA3* template in the reaction is shown. Error bars represent the standard error of the mean (SEM) between experiments.

Ino2 and Ino4 have been shown by gel-shift analysis of yeast extracts to bind directly to the UASINO in the *INO1* promoter (Lopes and Henry 1991; Ambroziak and Henry 1994; Bachhawat et al. 1995; Schwank et al. 1995). Binding was observed in extracts from cells grown under repressing or activating conditions, and was increased in the absence of Opi1 (Wagner et al. 1999). To monitor the interaction of Ino2 and Ino4 with the *INO1* promoter in vivo, we used chromatin immunoprecipitation (ChIP) (Solomon et al. 1988; Dedon et al. 1991). Consistent with the gel-shift experiments, we found that Ino2-HA and Ino4-HA bound to the *INO1* promoter under both repressing and activating conditions (Figure 2A). Real-time quantitative PCR analysis of immunoprecipitated DNA confirmed that both Ino2 and Ino4 associated with the *INO1* promoter constitutively (Figure 2B). Although we observed an increase in the association of Ino2 with the *INO1* promoter under inducing conditions compared with repressing conditions, these results argue that occupancy of the promoter by Ino2/Ino4 is not sufficient for activation but that it must be a subsequent step in the activation process that is regulated by the UPR.

**Figure 3:**
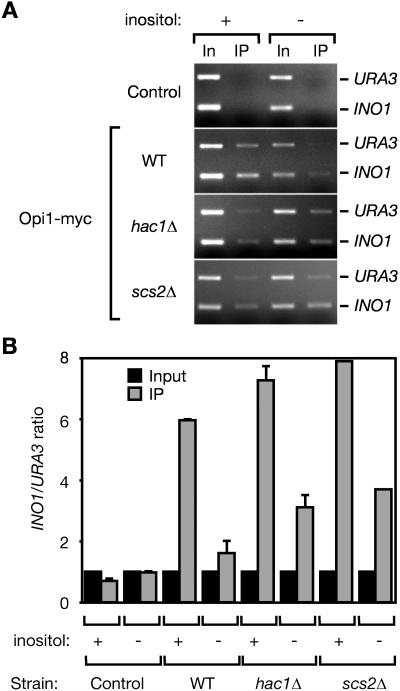
UPR-Dependent Dissociation of Opi1 from Chromatin (A) Chromatin-associated Opi1 dissociates upon activation of the UPR. Cells of the indicated genotypes were harvested after growth for 4.5 h with or without *myo*-inositol, fixed, and processed as in Figure 2. The *scs2*Δ mutant was transformed with pRS315-Opi1-*myc*, a *CEN ARS* plasmid that expresses Opi1-*myc* at endogenous levels. Input DNA (In) and immunoprecipitated DNA (IP) were analyzed by PCR using primers to amplify the *INO1* promoter and the *URA3* gene. Amplified DNA was separated by electrophoresis on ethidium bromide–stained agarose gels. (B) Quantitative PCR analysis. Input and IP fractions were analyzed by real-time quantitative PCR. The ratio of *INO1* promoter to *URA3* template in the reaction is shown. Error bars represent the SEM between experiments.

The molecular mechanism by which Opi1 represses transcription is not understood. In particular, it is not clear whether Opi1 binds to the *INO1* promoter directly. Early gel-shift experiments using yeast lysates suggested that Opi1 might interact with DNA (Lopes and Henry 1991). However, this association has not been confirmed, and its significance is unknown. We used ChIP analysis and real-time quantitative PCR to assess the interaction of Opi1 with the *INO1* promoter in vivo. We observed specific enrichment of the *INO1* promoter by immunoprecipitation of Opi1 from cells grown in the presence of inositol (repressing condition) but no significant enrichment of the *INO1* promoter by immunoprecipitation of Opi1 from cells starved for inositol (activating condition; Figure 3). By contrast, when we performed the immunoprecipitations from either *hac1*Δ or *scs2*Δ strains, we observed greater enrichment of the *INO1* promoter sequences from cells grown under both activating and repressing conditions. These results are consistent with the notion that Opi1 binds to chromatin at the *INO1* promoter and that the function of Hac1 and Scs2 is to promote Opi1 dissociation.

In contrast to immunoprecipitation of Ino2 and Ino4, which specifically recovered the *INO1* promoter and not the control *URA3* sequences (see Figure 2), immunoprecipitates of Opi1 recovered significant amounts of *URA3* sequences as well (Figure 3A, upper bands). It is clear from the quantitative PCR analysis that Opi1 binding to the *INO1* promoter is specific (Figure 3B). The different conditions used in the qualitative gel analysis (measuring PCR products after many cycles) and the quantitative PCR (measuring PCR products in the linear range of amplification) are likely to account for this difference.

### The *INO1* Gene Relocalizes within the Nucleus upon UPR Activation

**Figure 4:**
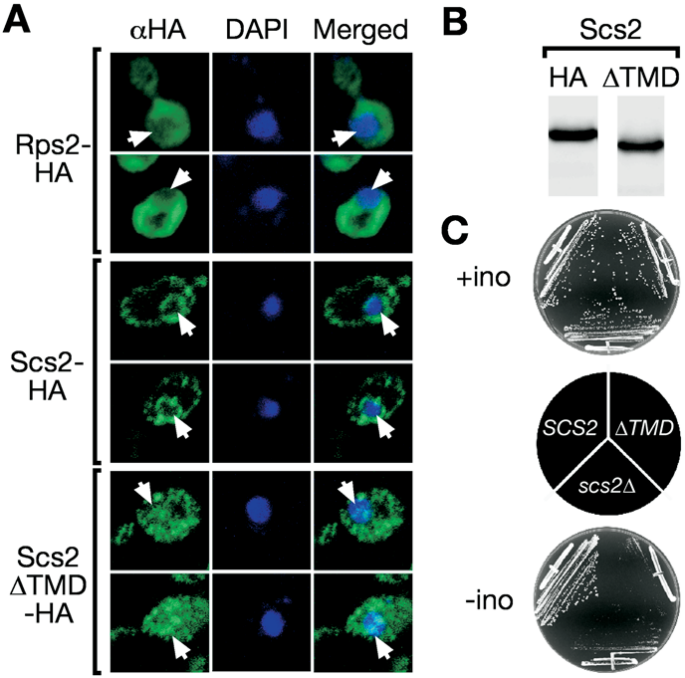
Membrane Association Is Essential for Scs2 Function The carboxyl-terminal transmembrane domain of Scs2 was removed by replacement with three copies of the HA epitope (Scs2ΔTMD-HA; Longtine et al. [1998]). (A) Scs2ΔTMD localization. Ribosomal protein S2 (Rps2-HA), Scs2-HA, and Scs2ΔTMD-HA were localized by immunofluorescence against the HA epitope. DNA was stained with 4′,6′-diamidino-2-phenylindole. Images were collected in a single z-plane (≤ 0.7 μm thick) by confocal microscopy. Unlike Rps2-HA, which was excluded from the nucleus (indicated with white arrows), Scs2ΔTMD-HA staining was uniform and evident in the nucleoplasm. (B) Scs2ΔTMD steady-state levels. Equal amounts of whole cell extract from cells expressing either Scs2-HA or Scs2ΔTMD-HA were analyzed by immunoblotting. (C) Scs2ΔTMD is nonfunctional. Strains expressing the indicated forms of Scs2 were streaked onto medium with or without *myo*-inositol and incubated for 2 d at 37 °C.

Since Opi1 dissociation from the *INO1* promoter correlates with activation and requires Hac1 and Scs2, an integral nuclear membrane protein, we wondered whether activation might occur at the nuclear periphery and thus might be dependent on the subnuclear positioning of the gene. Consistent with this hypothesis, we found that a form of Scs2 (Scs2ΔTMD) lacking the transmembrane domain, which was localized throughout the cell and was not excluded from the nucleus (Figure 4A, compare cytosolic protein Rps2 to Scs2ΔTMD for colocalization with 4′,6′-diamidino-2-phenylindole), and was nonfunctional, rendering cells inositol auxotrophs, despite being expressed at levels comparable to full length Scs2 (Figure 4B and 4C).

**Figure 5:**
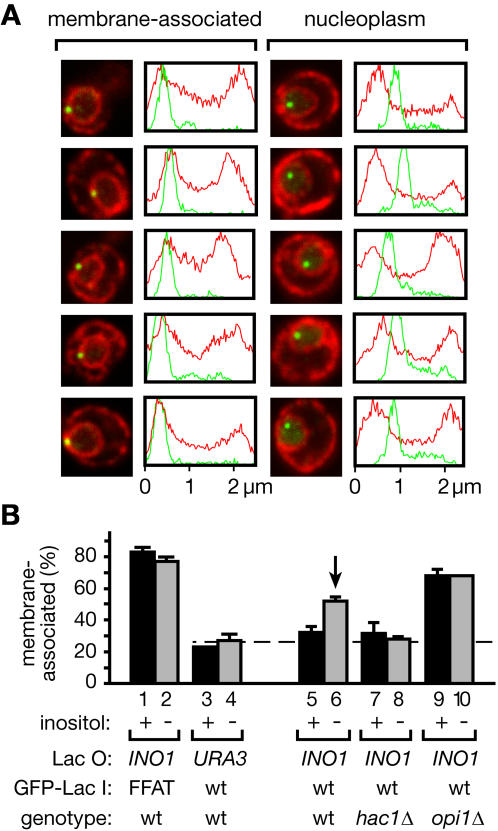
The *INO1* Gene Is Recruited to the Nuclear Membrane upon Activation An array of Lac operator repeats was integrated at *INO1* or *URA3* in strains expressing GFP-Lac repressor and *myc*-tagged Sec63. GFP-Lac repressor and Sec63-*myc* were localized in fixed cells by indirect immunofluorescence. Data were collected from single z sections representing the maximal, most focused signal from the Lac repressor. (A) Two classes of subnuclear localization. Shown are five representative examples of localization patterns that were scored as membrane-associated (photomicrographs and plots on left) or nucleoplasmic (right). For each image, the fluorescence intensity was plotted for each channel along a line that intersects both the Lac repressor spot and the center of the nucleus. (B) *INO1* is recruited to the nuclear membrane upon activation. The fraction of cells that scored as membrane-associated is plotted for each strain grown in the presence (+) or absence (–) of inositol. The site of integration of the Lac operator (Lac O), the version of the GFP-Lac repressor (GFP-Lac I; either wild-type or having the FFAT membrane-targeting signal) expressed, and the relevant genotype of each strain is indicated. The dashed line represents the mean membrane association of the *URA3* gene. The vertical arrow indicates the frequency of membrane association in the wild-type strain under activating conditions. Error bars represent the SEM between separate experiments. Each experiment scored at least 30 cells. The total number of cells (and experiments) scored for each column were: bar 1, 70 (2); bar 2, 66 (2); bar 3, 39 (1); bar 4, 71 (2); bar 5, 140 (4); bar 6, 88 (2); bar 7, 88 (2); bar 8, 92 (3); bar 9, 74 (2); and bar 10, 38 (1).

**Figure 6:**
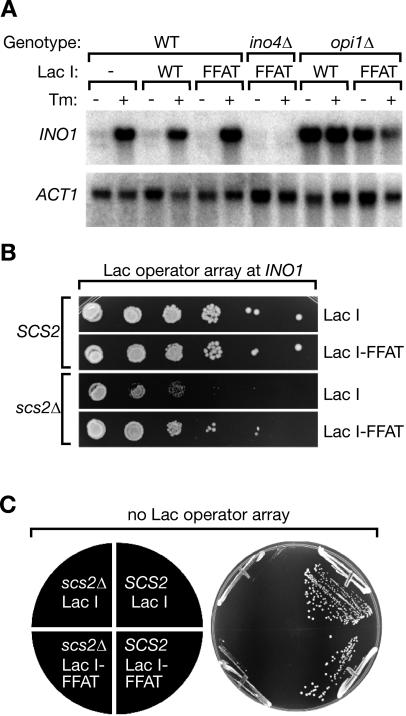
Artificial Relocalization of *INO1* Bypasses the Requirement for Scs2 (A) Northern blot analysis of membrane-targeted *INO1*. Strains of the indicated genotypes having the Lac operator array integrated at *INO1* and expressing either the wild-type GFP-Lac repressor or GFP-FFAT-Lac repressor were grown in the presence or absence of 1 μg/ml tunicamycin (Tm) for 4.5 h, harvested, and analyzed by Northern blot. Blots were probed for either *INO1* or *ACT1* (as a loading control) mRNA. The wild-type strain CRY1, lacking both the Lac operator array and the Lac repressor, was included in the first two lanes for comparison. (B) Wild-type or *scs2*Δ mutant strains in which the Lac operator had been integrated at *INO1* were transformed with either GFP-Lac repressor or GFP-FFAT-Lac repressor. The resulting transformants were serially diluted (tenfold between wells) and spotted onto medium lacking inositol, uracil, and histidine, and incubated for 2 d at 37 °C. (C) Wild-type and *scs2*Δ mutant strains transformed with either GFP-Lac repressor or GFP-FFAT-Lac repressor, but lacking the Lac operator, were streaked onto medium lacking inositol and histidine and incubated for 2 d at 37 °C.

If *INO1* were regulated at the nuclear periphery, then the *INO1* locus should colocalize with the nuclear membrane under activating conditions. To test this idea, we constructed a strain in which an array of Lac operator (Lac O in Figure 5) binding sites was integrated adjacent to the *INO1* locus (Robinett et al. 1996). The strain also expressed a green fluorescent protein (GFP)-Lac repressor fusion protein (GFP-Lac I in Figures 5 and 6) that binds to the Lac operator array to allow localization of the *INO1* gene. In a control strain, we integrated the same Lac operator array adjacent to the *URA3* locus. Cells were fixed and GFP was visualized by indirect immunofluorescence. Most cells showed a single intranuclear spot localizing the tagged gene; the remaining cells showed two spots due to their post-replication state in the cell cycle. In both the tagged *INO1* and the tagged *URA3* strains, we simultaneously visualized the ER and nuclear membrane by indirect immunofluorescence against Sec63-*myc* using a different fluorophore (Figure 5A).

To ask whether *INO1* associates with the nuclear membrane, we developed stringent criteria for scoring *INO1* localization (Figure 5A). Using confocal microscopy, we collected a single z slice through each cell that captured the brightest, most focused point of the GFP-visualized Lac operator array. Images in which this slice traversed the nucleus (i.e., cells that showed a clear nuclear membrane ring staining with a "hole" of nucleoplasm), were binned into two groups: Cells in which the peak of the spot corresponding to the tagged gene coincided with nuclear membrane staining were scored as membrane-associated, and cells in which the peak of the spot corresponding to the tagged gene was offset from nuclear membrane staining were scored as nucleoplasmic. This procedure allowed us to determine the fraction of cells in a given population in which the tagged gene colocalized with the membrane, thus providing a quantitative measure for membrane association. Five examples of each group, with fluorescence intensity plotted along a line bisecting the nucleus and the spot, are shown in Figure 5A.

To confirm that our scoring criterion would identify nuclear membrane association in a meaningful way, we applied it to two controls. As a control for membrane association, we localized *INO1* in a strain expressing GFP-Lac repressor fused to a peptide motif from Opi1 containing two phenylalanines in an acidic tract (FFAT motif), which serves as a nuclear membrane–targeting signal (Loewen et al. 2003). This motif was shown to bind to Scs2 and to be required for Opi1 targeting to the nuclear envelope (Loewen et al. 2003). Importantly, targeting of Opi1 to the nuclear membrane still occurred in the absence of Scs2 in an FFAT-dependent manner (Loewen et al. 2003), indicating that, in addition to Scs2, there must exist another, yet-unidentified receptor for FFAT in the nuclear membrane. As shown in Figure 5B, the localization of *INO1* scored as 85% membrane-associated (Figure 5B, bar 1), confirming both our scoring criteria and the previous result that FFAT indeed promotes nuclear membrane targeting.

As a control for random distribution, we localized *URA3* in a strain expressing GFP-Lac repressor without the FFAT targeting signal. *URA3* scored as 23% membrane-associated (Figure 5B, bar 3). Induction of the UPR after depletion of inositol had no effect on the localization of either FFAT-tagged *INO1* or *URA3* in these strains (Figure 5B, bars 2 and 4). Given that 25% of the volume of the nucleus is contained in the outer shell represented by only 10% of the radius, this level of background is consistent with a random distribution of the *URA3* gene throughout the nuclear volume. Based on the spatial resolution of our data (Figure 5A), a spot only 10% of the radius distant from the membrane signal would have been scored as membrane-associated. We therefore defined the mean frequency of membrane-association of the *URA3* control between these two conditions (25% ± 3%) as the baseline for subsequent comparisons (Figure 5B, dashed line).

We next compared the membrane association of *INO1* under repressing and activating conditions. Under repressing conditions, the membrane association of *INO1* was only slightly greater than the baseline (32% ± 3%; Figure 5B, bar 5). In striking contrast, when *INO1* was activated, the frequency of membrane association of *INO1* increased significantly over baseline (52% ± 3%; Figure 5B, bar 6). Thus, we conclude that, in a significant portion of cells, the *INO1* gene became associated with the nuclear membrane under UPR-inducing conditions.

To confirm that the observed recruitment was indeed due to UPR induction, we compared the membrane association of *INO1* under repressing or activating conditions in the *hac1*Δ mutant. Because Hac1 is required for activation of *INO1,* we predicted that membrane association would be prevented in this mutant. Indeed, *INO1* failed to become membrane associated in *hac1*Δ mutants starved for inositol (Figure 5B, bars 7 and 8). Our earlier experiments suggested that Hac1 functions to promote dissociation of Opi1 from the *INO1* promoter. We therefore tested next whether the presence of Opi1 prevents membrane association. To this end, we determined *INO1* localization in the *opi1*Δ strain, in which *INO1* is constitutively transcribed (Cox et al. 1997). Indeed, we observed a high degree of membrane association, both in the presence and absence of inositol (68% ± 5%; Figure 5B, bars 9 and 10).

### Artificial Recruitment of *INO1* Suppresses the *scs2*Δ Ino^–^ Phenotype

The experiments described above indicate that there is a correlation between membrane association of *INO1* and its transcriptional activation. To establish causality, we examined the effect of artificially targeting *INO1* to the nuclear membrane. In an otherwise wild-type background, artificial targeting of *INO1* to the nuclear membrane via FFAT-Lac repressor binding (same strain as in Figure 5B, bars 1 and 2) had no effect on *INO1* expression as assessed by Northern blot analysis (Figure 6A) or on the growth of the wild-type strain in the absence of inositol (Figure 6B; compare top two panels). This result suggests that membrane targeting per se is not sufficient to cause activation. In contrast, in the *scs2*Δ mutant we observed that the inositol-requiring growth phenotype of the strain was suppressed by expression of the membrane-targeted FFAT-Lac repressor (Figure 6B; compare bottom two panels). This effect was strictly dependent on having the Lac operator array integrated at the *INO1* locus; expressing GFP-FFAT-Lac repressor in the absence of the array (Figure 6C)—or if the array was integrated at the *URA3* locus (unpublished data)—did not improve the growth of the *scs2*Δ mutant in the absence of inositol. Consistent with the previous report that FFAT does not require Scs2 to promote nuclear membrane targeting, we observed approximately 50% membrane association of *INO1* in the strain expressing the FFAT-Lac repressor (78 cells counted, unpublished data). Thus, the defect in transcription of *INO1* in the *scs2*Δ mutant could be rescued, at least partially, through artificial targeting of *INO1* to the nuclear membrane. This result demonstrates that nuclear membrane association is functionally important for achieving *INO1* transcriptional activation.

## Discussion

It is becoming increasingly clear that the spatial arrangement of chromosomes within the nucleus is important for controlling the reactions that occur on DNA and might be regulated (reviewed in Cockell and Gasser [1999]; Isogai and Tjian [2003]). Here we have shown that activation of *INO1* occurs at the nuclear membrane and requires the integral membrane protein Scs2. Moreover, artificial recruitment of *INO1* to the nuclear membrane permits activation in the absence of Scs2, indicating that the precise intranuclear localization of a gene can profoundly influence its activation. Most importantly, we have shown that the localization of *INO1* depends on its activation state; gene recruitment therefore is a dynamic process and appears to play an important regulatory role.

### Regulation of Gene Localization

The nucleoplasm is bounded by the inner nuclear membrane, which provides a template that is likely to play a major role in organizing the genome. It is clear from numerous microscopic and biochemical studies that chromatin interacts with nuclear membrane proteins, associated proteins such as filamentous lamins, and nuclear pore complexes (DuPraw, 1965; Murray and Davies, 1979; Paddy, 1990; Worman et al., 1990; Belmont et al., 1993; Glass et al., 1993; Foisner and Gerace, 1993; Sukegawa and Blobel, 1993; Luderus et al., 1994; Marshall et al., 1996). Indeed, several transcriptionally regulated genes have been shown to colocalize with heterochromatin at the nuclear periphery when repressed (Csink and Henikoff 1996; Dernburg et al. 1996; Brown et al. 1997, 1999). Likewise, silencing of genes near telomeres requires physical tethering of telomeres to nuclear pore complexes at the nuclear periphery (Gotta et al. 1996; Maillet et al. 1996; Andrulis et al. 1998; Laroche et al. 1998; Galy et al. 2000; Andrulis et al. 2002; Feuerbach et al. 2002).

Thus, the nuclear periphery has been generally regarded as a transcriptionally repressive environment (Gotta et al. 1996; Maillet et al. 1996; Andrulis et al. 1998; Laroche et al. 1998; Galy et al. 2000; Andrulis et al. 2002; Feuerbach et al. 2002). In contrast, the work presented here shows that gene recruitment to the nuclear periphery can be important for transcriptional activation. This conclusion is supported by a recent study published while this manuscript was in preparation (Casolari et al. 2004). These authors found that a subset of actively transcribed genes associates with components of nuclear pore complexes and that activation of *GAL* genes correlates with their recruitment from the nucleoplasm to the nuclear periphery and pore-complex protein association (Casolari et al. 2004). The results presented here argue that recruitment of genes to the nuclear periphery is controlled by transcriptional regulators and is important for achieving transcriptional activation. Thus, together, the work by Casolari et al. (2004) and the work presented here demonstrate that gene recruitment to the nuclear periphery can have a general role in activating transcription.

This notion is consistent with the “gene gating hypothesis” put forward by Blobel (1985). As proposed in this hypothesis, transcription of certain genes may be obligatorily coupled to mRNA export through a particular nuclear pore complex. It remains to be shown for *INO1,* however, whether gene recruitment to the nuclear periphery involves interaction with nuclear pore complex components. Several other scenarios could explain why *INO1* activation might require gene recruitment to the nuclear periphery. First, *INO1* transcriptional activation requires the SAGA histone acetylase, and both the SWI/SNF and INO80 chromatin remodeling complexes (Kodaki et al. 1995; Pollard and Peterson 1997; Ebbert et al. 1999; Shen et al. 2000; Dietz et al. 2003). Conversely, repression requires the Sin3/Rpd3 histone deacetylase and the ISW chromatin remodeling complex (Hudak et al. 1994; Sugiyama and Nikawa 2001). Thus, if these factors have distinct subnuclear distributions, then the localization of genes regulated by them might influence their transcriptional state. Consistent with this notion, the SAGA complex interacts with nuclear pore complexes, and therefore might be concentrated at the nuclear periphery, where *INO1* activation occurs (Rodriguez-Navarro et al. 2004). Second, because *INO1* and many other UASINO-regulated genes are involved in the biosynthesis of phospholipids, it is possible that the state of the membrane itself plays a role, perhaps sensed by Scs2, in activating transcription. It has been shown that defects in phospholipids biosynthesis can disrupt regulation of *INO1,* although the mechanism of this regulation remains unknown (Greenberg et al. 1982; McGraw and Henry 1989; Griac et al. 1996; Griac 1997; Shirra et al. 2001). Third, inositol polyphosphates have been shown to regulate SWI/SNF-catalyzed chromatin remodeling, and it is possible that their production is spatially restricted (Shen et al. 2003; Steger et al. 2003).

### Role of Factors Regulating *INO1* Activation

**Figure 7:**
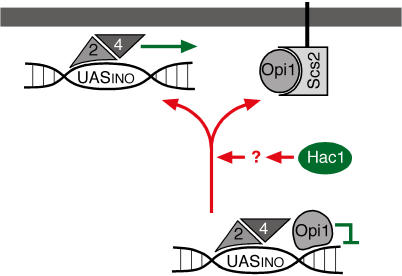
Model for *INO1* Gene Recruitment and Transcriptional Activation Ino2 and Ino4 bind constitutively to the *INO1* promoter. Under repressing conditions, Opi1 associates with chromatin to prevent activation, and the *INO1* locus localizes to the nucleoplasm. Hac1 synthesis under UPR-inducing conditions promotes dissociation of Opi1 from chromatin. Scs2 binds to Opi1 at the nuclear membrane to stabilize the non-chromatin-bound state. Dissociation is coupled to recruitment of *INO1* to the nuclear membrane, where transcriptional activation occurs.

Our current understanding of *INO1* activation is summarized in a model in Figure 7. The positive transcription activators Ino2 and Ino4 constitutively associate with the *INO1* promoter, which is kept transcriptionally repressed by Opi1. We do not currently understand the mechanism by which Opi1 prevents activation. Activation of the UPR leads to the production of Hac1, which, by an unknown mechanism, promotes Opi1 dissociation from chromatin. We propose that Scs2 at the nuclear membrane binds to Opi1 released from the DNA and thus keeps Opi1 sequestered and prevented from rebinding. Indeed, overproduction of Scs2 bypasses the requirement for Hac1 in activation of *INO1* transcription and allows *hac1*Δ cells to grow in the absence of inositol (Nikawa et al. 1995), supporting the role of Scs2 as a sink for Opi1 and suggesting that Opi1 may cycle between chromatin-bound and free states.

Both Hac1 (Cox et al. 1997) and Scs2 (see Figure 1) are dispensable for *INO1* activation in the absence of Opi1, suggesting that their role is to relieve Opi1 repression. However, our data suggest that Hac1 and Scs2 have distinct functions: While the absence of either protein prevents the dissociation of Opi1 from chromatin and the activation of *INO1,* we propose that the role of Hac1 is to promote dissociation and that of Scs2 is to prevent reassociation. This model explains why artificially tethering *INO1* to the nuclear membrane suppresses the absence of Scs2 but not the absence of Hac1 (unpublished data): We propose that the environment of membrane-tethered *INO1* promotes late steps in the transcription activation—such as chromatin remodeling, discussed above—permitting *INO1* to be expressed upon transient Hac1-induced Opi1 dissociation. Therefore, we envision that dissociation of Opi1 from the *INO1* promoter is coupled to the delivery of the gene to an environment near the nuclear membrane that is permissive for its activation.

The mechanistic role of Scs2 is currently not known. Its recently discovered function in promoting telomeric silencing (Craven and Petes 2001; Cuperus and Shore 2002) suggests that Scs2 may play a more global role in the regulation of transcription at the nuclear membrane. Scs2 contains a major sperm protein domain, named after a homologous protein in Ascaris suum sperm that forms a cytoskeletal structure and confers motility to sperm cells. It is thus tempting to speculate that Scs2 might similarly self-associate in the plane of the nuclear membrane, perhaps providing a two-dimensional matrix on which membrane-associated reactions could be organized. One suggestion from our data is that Scs2 may function as a local sink for Opi1. But it is also clear that other nuclear membrane components are likely to participate in the reaction. Opi1, for example, still localizes to nuclear membranes even in *scs2*Δ cells, indicating that another, yet-unidentified Opi1 binding partner must exist (Loewen et al. 2003; unpublished data). Similarly, artificial *INO1* recruitment to the membrane via the FFAT motif suppresses the *scs2*Δ phenotype (see Figure 6)—i.e., it is sufficient to position *INO1* in an environment permissive for its induction—yet the FFAT binding protein and the molecular nature of the permissive environment remain unknown.

Upon inducing the UPR, only 52% of the cells scored *INO1* as membrane-associated (see Figure 5). Thus, under activating conditions, two types of cells are present in the population at any one time: those in which the *INO1* gene is recruited to the membrane, and those in which the *INO1* gene is dispersed throughout the nucleoplasm. This score correlated with the level of *INO1* transcription; *INO1* was membrane-associated in 68% of the cells in the *opi1* mutant, which exhibits a correspondingly higher degree of activation than that observed in the wild-type strain. A quantitatively similar nuclear peripheral-nucleoplasmic distribution was observed upon activation of *GAL* genes (Casolari et al. 2004), suggesting that it may be a general feature of gene recruitment. There are at least two possible interpretations for the observed bimodal distributions. First, the distribution profiles might represent heterogeneity in the activation of *INO1* among cells. In this case, activation of *INO1* would be variable in individual cells exposed to identical conditions. Gene recruitment thus would stably trap *INO1* in a permissive environment for activation, and the localization of *INO1* would strictly correlate with its activation state. Alternatively, gene recruitment might alter the balance between two rapidly exchanging states; that is, stable membrane recruitment would not be required for activation. In this case, the observed distributions would represent snapshots of transient colocalization of *INO1* with the nuclear membrane within a population of cells that are uniformly activating transcription. Dynamic measurements of gene recruitment and single cell activity assays will need to be developed to distinguish between these possibilities. But no matter which of these possibilities proves to be correct, gene recruitment emerges as a new mechanism regulating eukaryotic gene expression and may be crucial to the regulation of many genes.

## Materials and Methods

### 

#### Antibodies and reagents

Monoclonal anti- HA antibody HA11 was obtained from Babco (Berkeley, California, United States). Monoclonal anti-*myc*, anti-*myc* agarose and anti-HA agarose were from Santa Cruz Biotechnology (Santa Cruz, California, United States). Monoclonal anti-Pgk1, rabbit polyclonal anti-GFP, goat anti mouse IgG-Alexafluor 594, and goat anti-rabbit IgG Alexafluor 488 were from Molecular Probes (Eugene, Oregon, United States).

All restriction endonucleases and DNA modification enzymes were from New England Biolabs (Beverly, Massachusetts, United States). Unless indicated otherwise, all other chemicals and reagents were from Sigma (St. Louis, Missouri, United States).

#### Strains and plasmids

All yeast strains used in this study were derived from wild-type strain CRY1 *(ade2–1 can1–100 his3–11,15 leu2–3,112 trp1–1 ura3–1 MAT*a*)*. Tags and disruptions marked with either the *kan^r^* gene from E. coli or the *His5* gene from S. pombe were introduced by recombination at the genomic loci as described (Longtine et al. 1998). Strains used in this study, with relevant differences indicated are JBY345 *(OPI1–13myc::kan^r^),* JBY350-r1 *(scs2*Δ*:: kan^r^),* JBY359 *(SCS2-HA:: kan^r^),* JBY356–1A *(opi1*Δ*::LEU2),* JBY356–1B *(opi1*Δ*::LEU2 scs2*Δ*:: kan^r^ ),* JBY356–1C *(scs2*Δ*:: kan^r^),* JBY356–1D (wild-type control), JBY361 *(scs2*Δ*TMD-HA:: kan^r^),* JBY370*(INO2-HA3::His5+),* JBY371 *(INO4-HA3::His5+),* JBY393 *(INO4-myc::His5+ MAT*a*),* JBY397 *(SEC63–13myc:: kan^r^ INO1:LacO128:URA3 HIS3:LacI-GFP),* JBY399 *(SEC63–13myc::Kan^r INO1:LacO128:URA3 HIS3:LacI-FFAT-GFP),* JBY401 *(ino4*Δ*::LEU2 SEC63–13myc::Kan^r^ INO1:LacO128:URA3 HIS3:LacI-GFP MATα),* JBY404 *(opi1*Δ*::LEU2 SEC63–13myc::Kan^r^ INO1:LacO128:URA3 HIS3:LacI-GFP),* JBY406 *(opi1*Δ*::LEU2 SEC63–13myc::Kan^r^ INO1:LacO128:URA3 HIS3:LacI-FFAT-GFP),* JBY409 *(SEC63–13myc::Kan^r^ URA3:LacO128:URA3 HIS3:LacI-GFP),* JBY412 *(INO2-myc::His5+),* JBY 416 *(hac1*Δ*::URA3 SEC63–13myc::Kan^r^ LacO128:INO1 HIS3:LacI-GFP)*.

Plasmid pRS315-Opi1-*myc* was created by first amplifying the *OPI1-myc* coding sequence and 686 bp upstream from the translational start site from strain JBY345 using the following primers: OPI1 promoter Up (5′-GGGAGATACAAACCATGAAG-3′) and OPI1 down (5′-ACTATACCTGAGAAAGCAACCTGACCTACAGG-3′). The resulting fragment was cloned into pCR2.1 using the Invitrogen (Carlsbad, California, United States) TOPO TA cloning kit. The *OPI1-myc* locus was then cloned into pRS315 as a HindIII-NotI fragment. Plasmid pASF144 expressing *GFP-lacI* has been described (Straight et al. 1996). Plasmid pGFP-FFAT-LacI was constructed by digesting pASF144 with EcoRI and ligating the fragment to the following hybridized oligonucleotides, encoding the FFAT motif from *OPI1:* LacI_FFAT1 (5′-AATTGGACGATGAGGAGTTTTTTGATGCCTCAGAGG-3′) and LacI_FFAT2 (5′-AATTCCTCTGAGGCATCAAAAAACTCCTCATCGTCC-3′). The orientation of the insert was confirmed by DNA sequencing. Both pAFS144 and pGFP-FFAT-LacI were digested with NheI, which cuts within the *HIS3* gene, and transformed into yeast.

Plasmid p6INO1LacO128 was constructed as follows. The *INO1* coding sequence, with 437 bp upstream and 758 bp downstream, was amplified from yeast genomic DNA using the following primers: INO1_promoter_Up (5′-GATGAGGCCGGTGCC-3′) and INO1_3′down (5′-AAGATTTCCTTCTTGGGCGC-3′), and cloned into pCR2.1 using the Invitrogen TOPO TA cloning kit, to produce pCR2.1-INO1. *INO1* was moved from pCR2.1 into pRS306 as a KpnI fragment, to produce pRS306-INO1. The Lac operator array was then cloned from pAFS52 into pRS306-INO1 as a HindIII-XhoI fragment, to produce plasmid 10.2. Because the Lac operator fragment was smaller than had been reported (2.5 kb instead of 10 kb), presumably reflecting loss of Lac operator repeats by recombination, the Lac operator array was duplicated by digesting plasmid 10.2 with HindIII and SalI and introducing a second copy of the 2.5-kb HindIII-XhoI fragment, as described (Robinett et al. 1996). The resulting plasmid, p6INO1LacO128, has a 5-kb Lac operator array, corresponding to approximately 128 repeats of the *lac* operator. To integrate this plasmid at *INO1,* p6INO1LacO128 was digested with BglII, which cuts within the *INO1* gene, and transformed into yeast.

The *INO1* gene was removed from this plasmid to generate p6LacO128. This plasmid was used to integrate the Lac operator array at *URA3* by digestion with StuI and transformation into yeast.

#### Immunoprecipitations

Cells were lysed using glass beads in IP buffer (50 mM Hepes-KOH pH 6.8, 150 mM potassium acetate, 2 mM magnesium acetate, and Complete Protease Inhibitors [Roche, Indianapolis, Indiana, United States]). The whole cell extract was used for coimmunoprecipitation of Ino2-*myc* and Ino4-HA. For immunoprecipitation of Opi1-*myc*, microsomes were pelleted by centrifugation for 10 min at 21,000 × *g* and resuspended in IP buffer. Triton X-100 was then added to either whole cell extract (Ino2-*myc*; final concentration of 1%) or the microsomal fraction (Opi1-*myc*; final concentration of 3%) and incubated for 30 min at 4 °C; detergent-insoluble material was then removed by centrifugation at 21,000 × *g*, 10 min. Anti-*myc* agarose was added to the supernatant and incubated 4 h at 4 °C, while rotating. For the experiment in Figure 1D, a fraction of the total was collected after antibody incubation. After agarose beads were pelleted, an equal fraction of the supernatant was collected. Beads were washed either five (see Figure 1B and 1D) or ten times (see Figure 1C) with IP buffer. A fraction of the final wash equal to the pellet fraction in Figure 1D was collected. After the final wash, proteins were eluted from the beads by heating in sample buffer and separating by SDS-PAGE (see Figure 1B and 1D). Trypsin digestion, gel extraction, and mass spectrometry of proteins that coimmunoprecipitated with Opi1 were performed by the HHMI Mass Spectrometry facility (University of California, Berkeley, United States).

#### Immunoblot and Northern blot analysis

For immunoblot analysis, 25 μg of crude protein, prepared using urea denaturing lysis buffer (Ruegsegger et al. 2001), was separated on Invitrogen NuPage polyacrylamide gels, transferred to nitrocellulose, and immunoblotted. RNA preparation, electrophoresis, and labeling of probes for Northern blot analysis has been described (Ruegsegger et al. 2001)**.**

#### Immunofluorescence

Immunofluorescence was carried out as described (Redding et al. 1991), except that cells were harvested and fixed by incubation in 100% methanol at –20 °C for 20 min. Fixed, spheroplasted, detergent-extracted cells were probed with 1:200 monoclonal anti-*myc* (see Figures 1 and 5), 1:200 monoclonal anti-HA (see Figure 4), or 1:1000 rabbit polyclonal anti-GFP (see Figure 5). Secondary antibodies were diluted 1:200. Vectashield mounting medium (Vector Laboratories, Burlingame, California, United States) was applied to cells before sealing slides and visualizing using a Leica TCS NT confocal microscope (Leica, Wetzlar, Germany). For experiments localizing the GFP-Lac repressor, we first collected a single z slice through each cell that captured the brightest, most focused point of the GFP-visualized Lac operator array. This z slice was picked blind with respect to the nuclear membrane staining. Images in which this slice showed a clear nuclear membrane ring staining with a "hole" of nucleoplasm were then scored as follows: Cells in which the peak of the GFP-Lac repressor spot coincided with Sec63-*myc* nuclear membrane staining were scored as membrane-associated, and cells in which the peak of this spot was offset from nuclear membrane staining were scored as nucleoplasmic.

#### Chromatin immunoprecipitation

Chromatin immunoprecipitation was carried out on strains expressing endogenous levels of tagged Ino2, Ino4, and Opi1 as described (Strahl-Bolsinger et al. 1997), with the following modifications. The time of formaldehyde fixation was specific for each tagged protein. Strains expressing Ino2-HA were fixed for 15 min, strains expressing Ino4-HA were fixed for 60 min, and strains expressing Opi1-*myc* were fixed for 30 min. After lysis, cells were sonicated 15 times for 10 s at 30% power using a microtip on a Vibracell VCX 600 Watt sonicator (Sonics and Materials, Newtown, Connecticut, United States). After sonication, lysates were centrifuged 10 min at 21,000 × *g* to remove insoluble material and incubated for 4 h with anti-HA agarose or anti-*myc* agarose. After elution of immunoprecipitated DNA and reversal of crosslinks by heating to 65 °C for 8 h, DNA was recovered using Qiaquick columns from Qiagen (Alameda, California, United States). Eluted samples were analyzed by PCR using the following primers against the *INO1* promoter or the *URA3* gene: INO1_proUp2 (5′-GGAATCGAAAGTGTTGAATG-3′), INO1_proDown (5′-CCCGACAACAGAACAAGCC-3′), URAup (5′- GGGAGACGCATTGGGTCAAC-3′), and URADown (5′-GTTCTTTGGAGTTCAATGCGTCC-3′).

#### Real time quantitative PCR analysis

PCR reactions were carried out as described (Rogatsky et al. 2003) using a DNA Engine Opiticon 2 Real-Time PCR machine (MJ Research, Waltham, Massachusetts, United States), using 1/25 of the immunoprecipitation fraction and an equal volume of a 1:400 dilution of the input fraction as template. Primers used were: INO1up3 5′-ATTGCCTTTTTCTTCGTTCC-3′), INO1down2 (5′-CATTCAACACTTTCGATTCC-3′), URAup2 (5′-AGACGCATTGGGTCAAC-3′), and URAdown2 (5′-CTTCCCTTTGCAAATAGTCC-3′). Dilution of the input fraction from 1:25 to 1:12,800 in fourfold steps demonstrated that reactions were within the linear range of template. This dilution series was used as a standard curve of C(T) values versus relative template concentration for both primer sets. The concentration of the *INO1* promoter and the *URA3* gene were calculated using this standard curve. The ratio of *INO1* promoter to *URA3* was corrected for each sample to make the input ratio equal to 1.0.

## Supporting Information

### Accession Numbers

The GenBank accession numbers of the genes and proteins discussed in this paper are Ire1 (NP_116622), *INO1* (NP_012382), Trl1 (NP_012448), Opi1 (NP_011843), Hac1 (NP_011946), Ino2 (NP_010408), Ino4 (NP_014533), Scs2 (NP_009461), Rap1 (NP_014183), *URA3* (NP_010893), *SSS1* (NP_010371), Pgk1 (NP_009938) *lacI* (NP_414879), and Ikaros (Q03267).

## Abbreviations

ChIP: chromatin immunoprecipitation
ER: endoplasmic reticulum
FFAT: ER/nuclear membrane targeting motif containing two phenylalanines in an acidic tract
GFP: green fluorescent protein
HA: hemagglutinin
SEM: standard error of the mean
UASINO: upstream activating sequence responsive to inositol
UPR: unfolded protein response
